# Supplementary material for: Observational study of haloperidol in hospitalized patients with COVID-19
Source: PLoS One. 2021 Feb 19;16(2):e0247122. doi: 10.1371/journal.pone.0247122 (PMC7895415; doi:10.1371/journal.pone.0247122)
Supplement: S1 Fig — (DOCX) [file pone.0247122.s001.docx]

**S1 Figure. Study cohort.**

17,076 patients with a positive COVID-19 RT-PCR test

who had been hospitalized for COVID-19 from January 24^th^ to May 1^st^

1,908 patients were excluded because of missing data or age:

- Hospitalization dates: N = 457

- Smoking status: N = 1,319

- Sex: N = 5

- Aged less than 18 years: N = 212

-

15,168 adult inpatients (86 received haloperidol and 15,082 did not)

47 patients who received haloperidol during the visit were excluded because the treatment started after study baseline (i.e. more than 48 hours from hospital admission) and/or after intubation or death

Exposed to haloperidol

N = 39

15,121 adult inpatients included in the propensity-matched and regression analyses

Not exposed to haloperidol

N = 15,082
