# Supplementary material for: Observational study of haloperidol in hospitalized patients with COVID-19
Source: PLoS One. 2021 Feb 19;16(2):e0247122. doi: 10.1371/journal.pone.0247122 (PMC7895415; doi:10.1371/journal.pone.0247122)
Supplement: S1 Table — (DOCX) [file pone.0247122.s002.docx]

**S1 Table. Associations of baseline clinical characteristics with the composite endpoint of intubation or death in the cohort of adult patients hospitalized for COVID-19 (N=15,121).**

|  | | |  |  |  | Composite endpoint of intubation or death |  |  |
| --- | --- | --- | --- | --- | --- | --- | --- | --- |
|  | | | Full sample (n=15,121) | With the endpoint (n=2,024) | Without the endpoint (n=13,097) | Crude analysis | Multivariable analysis |  |
|  | | | N (%) | N (%) | N (%) | HR (95% CI; p-value) | HR (95% CI; p-value) | Collinearity diagnostics (variance inflation factor) |
| *Characteristics* | | |  |  |  |  |  |  |
| Age | | |  |  |  |  |  | 1.07 |
| *18 to 57 years* | | | 7,620 (50.4%) | 316 (15.6%) | 7,304 (55.8%) | Ref. | Ref. |  |
| *More than 57 years* | | | 7,501 (49.6%) | 1,708 (84.4%) | 5,793 (44.2%) | 4.87 (4.31 - 5.49; <0.001*) | 3.13 (2.22 - 4.40; <0.001*) |  |
| Sex | | |  |  |  |  |  | 1.07 |
| *Women* | | | 7,950 (52.6%) | 723 (35.7%) | 7,227 (55.2%) | Ref. | Ref. |  |
| *Men* | | | 7,171 (47.4%) | 1,301 (64.3%) | 5,870 (44.8%) | 2.09 (1.91 - 2.29; <0.001*) | 1.69 (1.36 - 2.10; <0.001*) |  |
| Hospital | | |  |  |  |  |  | 1.04 |
| *AP-HP Centre – Paris University, Henri Mondor University Hospitals and at home hospitalization* | | | 7,029 (46.5%) | 650 (32.1%) | 6,379 (48.7%) | Ref. | Ref. |  |
| *AP-HP Nord and Hôpitaux Universitaires Paris Seine-Saint-Denis, Paris Saclay University and Sorbonne University* | | | 8,092 (53.5%) | 1,374 (67.9%) | 6,718 (51.3%) | 1.96 (1.78 - 2.15; <0.001*) | 1.12 (0.89 - 1.40; 0.340) |  |
| Obesity ^α^ | | |  |  |  |  |  | 1.03 |
|  | | Yes | 2,059 (13.6%) | 486 (24.0%) | 1,573 (12.0%) | 1.86 (1.68 - 2.07; <0.001*) | 1.32 (1.01 - 1.71; 0.042*) |  |
|  | | No | 13,062 (86.4%) | 1,538 (76.0%) | 11,524 (88.0%) |  |  |  |
| Smoking | | |  |  |  |  |  | 1.04 |
|  | Yes | | 1,293 (8.6%) | 294 (14.5%) | 999 (7.6%) | 1.60 (1.41 - 1.81; <0.001*) | 0.89 (0.63 - 1.26; 0.517) |  |
|  | No | | 13,828 (91.4%) | 1,730 (85.5%) | 12,098 (92.4%) | Ref. | Ref. |  |
| Any medical condition ^β^ | | |  |  |  |  |  | 1.29 |
| *Yes* | | | 4,116 (27.2%) | 1,401 (69.2%) | 2,715 (20.7%) | 7.09 (6.43 - 7.81; <0.001*) | 2.80 (2.22 - 3.54; <0.001*) |  |
| *No* | | | 11,005 (72.8%) | 623 (30.8%) | 10,382 (79.3%) | Ref. | Ref. |  |
| Any medication according to compassionate use or as part of a clinical trial | | |  |  |  |  |  | 1.09 |
| *Yes* | | | 1,901 (12.6%) | 527 (26.0%) | 1,374 (10.5%) | 2.38 (2.15 - 2.63; <0.001*) | 1.13 (0.95 - 1.35; 0.165) |  |
| *No* | | | 13,220 (87.4%) | 1,497 (74.0%) | 11,723 (89.5%) | Ref. | Ref. |  |
| Any current psychiatric disorder or delirium ^¥^ | | |  |  |  |  |  | 1.28 |
| *Yes* | | | 945 (6.3%) | 412 (20.4%) | 533 (4.07%) | 3.71 (3.32 - 4.13; <0.001*) | 1.33 (1.06 - 1.68; 0.015*) |  |
| *No* | | | 14,176 (93.8%) | 1,612 (79.6%) | 12,564 (95.9%) | Ref. | Ref. |  |
| Any antipsychotic (other than haloperidol) | | |  |  |  |  |  | 1.14 |
| *Yes* | | | 485 (3.2%) | 156 (7.7%) | 329 (2.5%) | 2.24 (1.9 - 2.64; <0.001*) | 0.96 (0.69 - 1.32; 0.787) |  |
| *No* | | | 14,636 (96.8%) | 1,868 (92.3%) | 12,768 (97.5%) | Ref. | Ref. |  |
| Any other psychotropic medication ^Ω^ | | |  |  |  |  |  | 1.24 |
| *Yes* | | | 2,200 (14.5%) | 744 (36.8%) | 1456 (11.1%) | 3.05 (2.79 - 3.34; <0.001*) | 1.49 (1.24 - 1.80; <0.001*) |  |
| *No* | | | 12,921 (85.5%) | 1,280 (63.2%) | 11,641 (88.9%) | Ref. | Ref. |  |

^α^ Defined as having a body-mass index higher than 30 kg/m^2^ or based on ICD-10 codes (E66.0, E66.1, E66.2, E66.8, E66.9).

^β^ Included diabetes milletus (E11), diseases of the circulatory system (I00-I99), diseases of the respiratory system (J00-J99), neoplasms (C00-C96), and diseases of the blood and blood-forming organs and certain disorders involving the immune mechanism (D5-D8) based on ICD-10 codes.

^¥^ Assessed using ICD-10 codes (F00-F99 or R41.0).

^Ω^ Included any antidepressant, benzodiazepine, Z-drug, or mood stabilizer (i.e., lithium or antiepileptic medications with mood stabilizing effects).

* p-value is significant (p<0.05)

Abbreviations: HR, hazard ratio; CI, confidence interval.
