# Supplementary material for: Observational study of haloperidol in hospitalized patients with COVID-19
Source: PLoS One. 2021 Feb 19;16(2):e0247122. doi: 10.1371/journal.pone.0247122 (PMC7895415; doi:10.1371/journal.pone.0247122)
Supplement: S2 Table — (DOCX) [file pone.0247122.s003.docx]

**S2 Table. Associations of baseline clinical characteristics with the endpoint of discharge home in the cohort of adult patients hospitalized for COVID-19 who survived (N=11,572).**

|  |  |  |  | Endpoint of discharge home among survivors |  |  |
| --- | --- | --- | --- | --- | --- | --- |
|  | Full sample (n=11,572) | With the endpoint (n=9,923) | Without the endpoint (n=12,760) | Crude analysis | Multivariable analysis |  |
|  | N (%) | N (%) | N (%) | HR (95% CI; p-value) | HR (95% CI; p-value) | Collinearity diagnostics (variance inflation factor) |
| *Characteristics* |  |  |  |  |  |  |
| Age |  |  |  |  |  | 1.06 |
| *18 to 57 years* | 7,026 (60.7%) | 6,629 (66.8%) | 397 (24.1%) | Ref. | Ref. |  |
| *More than 57 years* | 4,546 (39.3%) | 3,294 (33.2%) | 1,252 (75.9%) | 0.38 (0.37 - 0.4; <0.001*) | 0.70 (0.68 - 0.73; <0.001*) |  |
| Sex |  |  |  |  |  | 1.04 |
| *Women* | 6,291 (54.4%) | 5,626 (56.7%) | 665 (40.3%) | Ref. | Ref. |  |
| *Men* | 5,281 (45.6%) | 4,297 (43.3%) | 984 (59.7%) | 0.68 (0.66 - 0.71; <0.001*) | 0.94 (0.91 - 0.96; <0.001*) |  |
| Hospital |  |  |  |  |  | 1.03 |
| *AP-HP Centre – Paris University, Henri Mondor University Hospitals and at home hospitalization* | 5,862 (50.7%) | 5,379 (54.2%) | 483 (29.3%) | Ref. | Ref. |  |
| *AP-HP Nord and Hôpitaux Universitaires Paris Seine-Saint-Denis, Paris Saclay University and Sorbonne University* | 5,710 (49.3%) | 4,544 (45.8%) | 1,166 (70.7%) | 0.49 (0.47 - 0.51; <0.001*) | 0.75 (0.73 - 0.77; <0.001*) |  |
| Obesity ^α^ |  |  |  |  |  | 1.03 |
| *Yes* | 1,354 (11.7%) | 1,028 (10.4%) | 326 (19.8%) | 0.6 (0.56 - 0.64; <0.001*) | 0.90 (0.85 - 0.95; <0.001*) |  |
| *No* | 10,218 (88.3%) | 8,895 (89.6%) | 1,323 (80.2%) | Ref. | Ref. |  |
| Smoking |  |  |  |  |  | 1.04 |
| *Yes* | 796 (6.9%) | 588 (5.9%) | 208 (12.6%) | 0.61 (0.56 - 0.66; <0.001*) | 0.96 (0.90 - 1.03; 0.261) |  |
| *No* | 10,776 (93.1%) | 9,335 (94.1%) | 1,441 (87.4%) | Ref. | Ref. |  |
| Any medical conditions ^β^ |  |  |  |  |  | 1.13 |
| *Yes* | 2,820 (24.4%) | 1,763 (17.8%) | 1,057 (64.1%) | 0.23 (0.22 - 0.25; <0.001*) | 0.26 (0.24 - 0.27; <0.001*) |  |
| *No* | 8,752 (75.6%) | 8,160 (82.2%) | 592 (35.9%) | Ref. | Ref. |  |
| Any medication according to compassionate use or as part of a clinical trial |  |  |  |  |  | 1.06 |
| *Yes* | 1,441 (12.5%) | 978 (9.9%) | 463 (28.1%) | 0.35 (0.33 - 0.37; <0.001*) | 0.42 (0.38 - 0.45; <0.001*) |  |
| *No* | 10,131 (87.5%) | 8,945 (90.1%) | 1,186 (71.9%) | Ref. | Ref. |  |
| Any current psychiatric disorder or delirium ^¥^ |  |  |  |  |  | 1.11 |
| *Yes* | 551 (4.8%) | 250 (2.5%) | 301 (18.3%) | 0.24 (0.21 - 0.27; <0.001*) | 0.62 (0.52 - 0.72; <0.001*) |  |
| *No* | 11,021 (95.2%) | 9,673 (97.5%) | 1,348 (81.7%) | Ref. | Ref. |  |
| Any antipsychotic (other than haloperidol) |  |  |  |  |  | 1.10 |
| *Yes* | 315 (2.7%) | 153 (1.5%) | 162 (9.8%) | 0.27 (0.23 - 0.32; <0.001*) | 0.54 (0.42 - 0.70; <0.001*) |  |
| *No* | 11,257 (97.3%) | 9,770 (98.5%) | 1,487 (90.2%) | Ref. | Ref. |  |
| Any other psychotropic medication ^Ω^ |  |  |  |  |  | 1.13 |
| *Yes* | 1,359 (11.7%) | 777 (7.8%) | 582 (35.3%) | 0.28 (0.26 - 0.3; <0.001*) | 0.42 (0.38 - 0.47; <0.001*) |  |
| *No* | 10,213 (88.3%) | 9,146 (92.2%) | 1,067 (64.7%) | Ref. | Ref. |  |

^α^ Defined as having a body-mass index higher than 30 kg/m^2^ or based on ICD-10 codes (E66.0, E66.1, E66.2, E66.8, E66.9).

^β^ Included diabetes milletus (E11), diseases of the circulatory system (I00-I99), diseases of the respiratory system (J00-J99), neoplasms (C00-C96), and diseases of the blood and blood-forming organs and certain disorders involving the immune mechanism (D5-D8) based on ICD-10 codes.

^¥^ Assessed using ICD-10 codes (F00-F99 or R41.0).

^Ω^ Included any antidepressant, benzodiazepine, Z-drug, or mood stabilizer (i.e., lithium or antiepileptic medications with mood stabilizing effects).

* p-value is significant (p<0.05)

Abbreviations: HR, hazard ratio; CI, confidence interval.
