# Supplementary material for: Observational study of haloperidol in hospitalized patients with COVID-19
Source: PLoS One. 2021 Feb 19;16(2):e0247122. doi: 10.1371/journal.pone.0247122 (PMC7895415; doi:10.1371/journal.pone.0247122)
Supplement: S4 Table — (DOCX) [file pone.0247122.s005.docx]

**S4 Table. Associations between haloperidol use and the endpoints of intubation or death and discharge home among survivors, in the full sample and in the matched analytic sample of patients hospitalized for COVID-19, when considering the variable hospital as a random effects variable.**

|  | **Intubation or death** | **Discharge home  among survivors** |
| --- | --- | --- |
| ***Full sample*** |  |  |
| Number of events /  Number of patients (%) | 2,024 / 15,121 (13.4%) | 9,923 / 11,572 (85.8%) |
| *Haloperidol* | 9 / 39 (23.1%) | 16 / 26 (61.5%) |
| *No haloperidol* | 2,015 / 15,082 (13.4%) | 9,907 / 11,546 (85.8%) |
| Analysis adjusted for hospital  HR (95% CI; p-value)^α^ | 1.49 (0.77 – 2.87; 0.230) | 0.41 (0.25 – 0.67; <0.001*) |
| Multivariable analysis  HR (95% CI; p-value) ^α^ | 0.78 (0.40 – 1.50; 0.450) | 1.01 (0.61 – 1.65; 0.970) |
| Propensity score analysis with inverse probability weighting  HR (95% CI; p-value) ^α^ | 1.30 (0.70 – 2.39; 0.410) | 1.10 (0.66 – 1.81; 0.720) |
| ***Matched analytic sample*** |  |  |
| Number of events /  Number of patients (%) | 59 / 195 (30.3%) | 65 / 130 (50.0%) |
| *Haloperidol* | 9 / 39 (23.1%) | 16 / 26 (61.5%) |
| *No haloperidol* | 50 / 156 (32.1%) | 49 / 104 (47.1%) |
| Crude analysis  HR (95% CI; p-value) | 0.76 (0.37 – 1.55; 0.450) | 1.43 (0.82 – 2.52; 0.210) |

^α^ Adjusted for hospital considered as a random effects variable

* p-value is significant (p<0.05)

Abbreviations: HR, hazard ratio; CI, confidence interval.
