# Supplementary material for: Observational study of haloperidol in hospitalized patients with COVID-19
Source: PLoS One. 2021 Feb 19;16(2):e0247122. doi: 10.1371/journal.pone.0247122 (PMC7895415; doi:10.1371/journal.pone.0247122)
Supplement: S5 Table — (DOCX) [file pone.0247122.s006.docx]

**S5 Table. Associations between haloperidol use and the endpoints of intubation or death and discharge home among survivors, in the full sample and in the matched analytic sample of patients hospitalized for COVID-19, following imputation of missing data with multiple imputation.**

|  | **Intubation or death** | **Discharge home  among survivors** |
| --- | --- | --- |
| ***Full sample*** |  |  |
| Number of events /  Number of patients (%) | 2,482 / 16 865 (14.7%) | 11,880 / 15,090 (78.7%) |
| *Haloperidol* | 10 / 40 (25.0%) | 19 / 32 (59.4%) |
| *No haloperidol* | 24,472 / 16,825 (14.7%) | 11,861 / 15,058 (78.8%) |
| Crude analysis  HR (95% CI; p-value) | 1.75 (0.94 – 3.25; 0.078) | 0.24 (0.12 – 0.50; <0.001*) |
| Multivariable analysis  HR (95% CI; p-value) | 0.61 (0.19 – 1.98; 0.411) | 0.95 (0.57 – 1.61; 0.842) |
| Propensity score analysis with inverse probability weighting  HR (95% CI; p-value) | 1.38 (0.75 – 2.52; 0.296) | 1.24 (0.78 – 2.02; 0.347) |
| ***Matched analytic sample*** |  |  |
| Number of events /  Number of patients (%) | 61 / 200 (30.5%) | 73 / 160 (45.6%) |
| *Haloperidol* | 10 / 40 (25.0%) | 19 / 32 (59.4%) |
| *No haloperidol* | 51 / 160 (31.9%) | 54 / 128 (42.2%) |
| Crude analysis  HR (95% CI; p-value) | 0.88 (0.45 – 1.75 (0.724) | 1.59 (0.94 – 2.68; 0.084) |

* p-value is significant (p<0.05)

Abbreviations: HR, hazard ratio; CI, confidence interval.
