# Supplementary material for: Observational study of haloperidol in hospitalized patients with COVID-19
Source: PLoS One. 2021 Feb 19;16(2):e0247122. doi: 10.1371/journal.pone.0247122 (PMC7895415; doi:10.1371/journal.pone.0247122)
Supplement: S7 Table — (DOCX) [file pone.0247122.s008.docx]

**S7 Table. Comparing haloperidol use only during the three months before hospitalization versus only during the visit for the endpoints of intubation or death and discharge home among survivors.**

|  | **Full sample** | | | | **Matched analytic samples** | |
| --- | --- | --- | --- | --- | --- | --- |
|  | **Number of events / Number of patients in the exposed groups** | **Crude Cox regression analysis** | **Multivariable Cox regression analysis** | **Analysis weighted by inverse-probability-weighting weights** | **Number of events / Number of patients** | **Univariate Cox regression in matched analytic samples** |
|  | N / % | HR  (95% CI; p-value) | HR  (95% CI; p-value) | HR  (95% CI; p-value) | N / % | HR  (95% CI; p-value) |
| **Intubation or death** |  |  |  |  |  |  |
| No exposure during the hospitalization and in the past 3 months | 1998 / 15032 (13.3) | 0.48 (0.22 – 1.09; 0.078) | 0.85 (0.39 – 1.85; 0.685) | 0.76 (0.33 – 1.74; 0.510) | 8 / 25 (32.0) | 1.17 (0.39 – 3.49; 0.782) |
| Haloperidol in the past 3 months but not during the hospitalization | 17 / 50 (34.0) | 0.74 (0.28 – 1.94; 0.534) | 0.91 (0.23 – 3.59 ; 0.899) | 0.70 (0.22 – 2.26 ; 0.549) | 8 / 25 (32.0) | 0.58 (0.18 – 1.82 ; 0.348) |
| Haloperidol during the hospitalization | 6 / 25 (24.0) | Ref. | Ref. | Ref. | 6 / 25 (24.0) | Ref. |
| **Discharge home** |  |  |  |  |  |  |
| No exposure during the hospitalization and in the past 3 months | 9900 / 11536 (85.8) | 3.86 (2.00 – 7.47 ; <0.001*) | 0.86 (0.43 – 1.71 ; 0.670) | 1.09 (0.55 – 2.15 ; 0.798) | 8 / 16 (56.2) | 0.89 (0.34 – 2.32 ; 0.814) |
| Haloperidol in the past 3 months but not during the hospitalization | 7 / 10 (70.0) | 2.42 (0.92 – 6.32; 0.073) | 2.30 (0.66 – 7.96; 0.190) | 2.35 (0.44 – 12.66; 0.319) | 6 / 10 (60.0) | 1.75 (0.55 – 5.55; 0.341) |
| Haloperidol during the hospitalization | 9 / 16 (56.2) | Ref. | Ref. | Ref. | 9 / 16 (56.2) | Ref. |

* p-value is significant (p<0.05).

Abbreviations: HR, hazard ratio; CI, confidence interval.
