# Supplementary material for: Observational study of haloperidol in hospitalized patients with COVID-19
Source: PLoS One. 2021 Feb 19;16(2):e0247122. doi: 10.1371/journal.pone.0247122 (PMC7895415; doi:10.1371/journal.pone.0247122)
Supplement: S8 Table — (DOCX) [file pone.0247122.s009.docx]

**S8 Table. Association between haloperidol dose and the endpoint of intubation or death.**

|  |  | | **Full sample** | | | **Matched analytic samples** | |
| --- | --- | --- | --- | --- | --- | --- | --- |
|  | **Number of events / Number of patients in the exposed groups** | **Crude Cox regression analysis** | | **Multivariable Cox regression analysis**^β^ | **Analysis weighted by inverse-probability-weighting weights**^β^ | **Number of events / Number of patients in the matched control groups**^β^ | **Univariate Cox regression in a matched analytic sample** |
|  | N / % | HR (95% CI; p-value) | | HR (95% CI; p-value) | HR (95% CI; p-value) | N / % | HR (95% CI; p-value) |
| Haloperidol (n=31) |  |  | |  |  |  |  |
| *Low dose* | 5 / 19 (26.3%) | Ref. | | Ref. | Ref. | 3 / 12 (25.0%) | Ref. |
| *High dose* ^α^ | 3 / 12 (25.0%) | 0.73 (0.18 – 3.25; 0.722) | | 0.60 (0.18 – 1.99; 0.407) | 0.40 (0.07 – 2.40; 0.317) | 3 / 12 (25.0%) | 0.70 (0.11 – 4.25; 0.696) |

Information was lacking to confidently assess haloperidol doses in 8 patients (20.5%), who were excluded from these analyses.

^α^ High dose was defined as having a dose equal or higher than the median of values in patients receiving haloperidol (i.e., 3 mg).

^β^ Adjusted for sex, age, and any medical condition.

Abbreviations: HR, hazard ratio; CI, confidence interval.
