# Supplementary material for: Observational study of haloperidol in hospitalized patients with COVID-19
Source: PLoS One. 2021 Feb 19;16(2):e0247122. doi: 10.1371/journal.pone.0247122 (PMC7895415; doi:10.1371/journal.pone.0247122)
Supplement: S9 Table — (DOCX) [file pone.0247122.s010.docx]

**S9 Table. Association between haloperidol dose and the endpoint of discharge home among survivors.**

|  |  | | **Full sample** | | | **Matched analytic samples** | |
| --- | --- | --- | --- | --- | --- | --- | --- |
|  | **Number of events / Number of patients in the exposed groups** | **Crude Cox regression analysis** | | **Multivariable Cox regression analysis**^β^ | **Analysis weighted by inverse-probability-weighting weights** ^β^ | **Number of events / Number of patients in the matched control groups**^β^ | **Univariate Cox regression in a matched analytic sample** |
|  | N / % | HR (95% CI; p-value) | | HR (95% CI; p-value) | HR (95% CI; p-value) | N / % | HR (95% CI; p-value) |
| Haloperidol (n=29) |  |  | |  |  |  |  |
| *Low dose* | 7 / 13 (53.8%) | Ref. | | Ref. | Ref. | 4 / 8 (50.0%) | Ref. |
| *High dose* ^α^ | 4 / 8 (50.0%) | 0.91 (0.26 – 3.13; 0.876) | | 2.19 (0.41 – 11.62; 0.357) | 1.77 (0.38 – 8.37; 0.469) | 4 / 8 (50.0%) | 1.16 (0.29 – 4.64; 0.838) |

Information was lacking to confidently assess haloperidol doses in 10 patients (34.5%), who were excluded from these analyses.

^α^ High dose was defined as having a dose equal or higher than the median of values in patients receiving haloperidol (i.e., 3 mg).

^β^ Adjusted for sex, age, and any medical condition.

Abbreviations: HR, hazard ratio; CI, confidence interval.
