# Supplementary material for: Observational study of haloperidol in hospitalized patients with COVID-19
Source: PLoS One. 2021 Feb 19;16(2):e0247122. doi: 10.1371/journal.pone.0247122 (PMC7895415; doi:10.1371/journal.pone.0247122)
Supplement: Attachment — Submitted filename: plos one review --2020 1023 revised.docx [file pone.0247122.s011.docx]

The Authors examined the treatment effect of haloperidol use in hospitalized COVID-19 patients at the APHP Greater Paris University hospitals. The primary endpoint was a composite of intubation or death and the secondary endpoint was discharge to home among survivors in time-to-event analyses. To control selection bias due to nonrandom assignment of treatments, the authors built a propensity score model with demographic and clinical factors and medication uses as predictors for haloperidol use. The authors then calculated stabilized weights and applied the weights to a multivariable Cox regression model with the start time being the date of hospital admission. Sensitivity analyses were also conducted including a multivariable Cox regression model with the same variables as in the propensity score model and a univariate Cox regression model in a matched analytic sample. The manuscript was well organized and clearly written. My major concerns are:

1. For those who received the treatment not on admission date but during hospitalization, their follow-up periods consisted of two parts: untreated and treated. The authors did not consider these two different follow-up periods in their analyses.
2. Related to comment 1, immortal bias may arise because patients had to be alive before receiving the treatment compared to the control group who could die any time after hospital admission.
3. It does not seem right to include medications prescribed during hospitalization in propensity score model. This is because these medications could be given after patients received haloperidol treatment.
4. Table 1 shows that after applying weighting using IPW, several variables were still significantly different between the two groups. I was wondering if the authors explored higher orders and interactions between covariates to improve the propensity score model. Another way is to include these unbalanced covariates in the Cox model.
5. In line 173, the authors mentioned ‘weighted Cox regression models were used when proportional hazards were not met’. I am not aware of such approach for handling the nonproportional hazard issue. Please elaborate this approach and cite the reference(s).
6. I recommend including those who did not consent in Figure 1.
7. I am not sure if the linear form of the dosage is appropriate in the Cox model. I was wondering if the authors had tried different functional forms for this variable when studying the dosage effect.
8. The power estimation did not mean much here because weighting was not considered.
