# Supplementary material for: Observational study of haloperidol in hospitalized patients with COVID-19
Source: PLoS One. 2021 Feb 19;16(2):e0247122. doi: 10.1371/journal.pone.0247122 (PMC7895415; doi:10.1371/journal.pone.0247122)
Supplement: Attachment — Submitted filename: Response to Reviewers.docx [file pone.0247122.s013.docx]

Manuscript PONE-D-20-30929-R2, "Observational Study of Haloperidol in Hospitalized Patients with COVID-19",

January 18^th^, 2020,

Wisit Cheungpasitporn, M.D.,

Editor, *PLOS ONE*

Dear Dr. Cheungpasitporn,

Thank you for the opportunity to submit a revised version of manuscript PONE-D-20-30929-R1, "Observational Study of Haloperidol in Hospitalized Patients with COVID-19". We want to thank the reviewers for their helpful comments, which have allowed us to strengthen the manuscript. A point-by-point response is included below, and the corresponding changes have been made in the manuscript and have been highlighted in yellow.

The revised manuscript contains no data, patient information, or other material or results that have been published or are in press or submitted elsewhere.

We look forward to hearing from you and thank you in advance for considering this contribution.

Competing Interests Statement

We have read the journal's policy and the authors of this manuscript have the following competing interests: NH has received personal fees and non-financial support from Lundbeck, outside the submitted work. FL has received speaker and consulting fees from Janssen-Cilag, Euthérapie-Servier, and Lundbeck, outside the submitted work. CL reports personal fees and non-financial support from Janssen-Cilag, Lundbeck, Otsuka Pharmaceutical, and Boehringer Ingelheim, outside the submitted work. GA reports personal fees from Pfizer, Pierre Fabre and Lundbeck, outside the submitted work. Other authors declare no competing interests. This does not alter our adherence to PLOS ONE policies on sharing data and materials. The data that support the findings of this study are available from the AP-HP Health Data Warehouse (Entrepôt de Données de Santé (EDS)). Restrictions apply to the availability of these data, which were used under license for this study. Data are available at https://eds.aphp.fr// with the permission of AP-HP Health Data Warehouse (Entrepôt de Données de Santé (EDS)).

Best Regards,

Nicolas Hoertel, M.D., M.P.H., Ph.D.,

On behalf of all authors and of AP-HP / Universities / INSERM Covid-19 research collaboration and AP-HP Covid CDR Initiative

**Editor**

**1/ The reviewers have still raised several points which we believe additional modifications are necessary to improve the manuscript, taking into account the reviewers' remarks. Please consider and address each of the comments raised by the reviewers.**
Answer: We thank the reviewers for these additional helpful comments, which have allowed us to strengthen the manuscript. A point-by-point response is included below, and the corresponding changes have been made in the manuscript and have been highlighted in yellow in a separate file.

**Reviewer #1**

**2/ The authors have replied in a very detailed and successful way to previous comments. The revised version of the article is much improved.**
Answer: We thank the Reviewer for this positive comment.

**3/ I noted some inconsistent data reported in TABLE 1. The authors have revised their article so that the matched analysis is now the primary analysis. As such, they have stated that the matching was successful in balancing the exposed haloperidol persons (N=39) and referent matched non-haloperidol persons (N=156). There appears to be some issue with the reported balancing in TABLE 1 between these groups (exposed versus matched unexposed). The reported matching on age and sex seem reasonable. However, the reported matching seems to be flipped or incorrectly reported in TABLE 1 for smoking yes (28.2% vs. 71.8%) for obesity yes (23.1% vs. 76.9%) and for the medical conditions and other medications used (the last 5 characteristics in TABLE 1). Please ensure that there has not be some accidental number transposition for these characteristics in column 1 (exposed haloperidol N=39) and column 3 (non-exposed matched N=156).**

Answer: We warmly thank the Reviewer for noticing this accidental number transposition, which is now corrected (see Table 1).

**Reviewer #2**

**4/ Please soften the conclusions stated on lines 69 and 387. The data and analyses support only the weaker conclusion that no association was detected.**

Answer: Following this comment, we softened these two conclusion sentences as follows:

P4, L68-71: “Findings from this multicenter observational study suggest that haloperidol use prescribed at a mean dose of 4.5 mg per day (SD=5.2) for a mean duration of 8.4 days (SD=7.2) may not be associated with risk of intubation or death, or with time to discharge home, among adult patients hospitalized for COVID-19.”

P20, L386-388: “Findings form this multicenter observational study suggest that haloperidol prescribed at a mean daily dose of 4.5 mg per day (SD=5.2) for a mean duration of 8.4 days (SD=7.2) may not be associated with risk of intubation or death, or with time to discharge home, among patients hospitalized for COVID-19.”

**Reviewer #3**

**5/ The authors addressed all the reviewers concerns and did a great job. I only have a few minor suggestions.**

Answer: We thank the Reviewer for this positive comment.

**6/ In the result sections**

**1. Characteristics of the cohort. I would suggest replacing the mean by median and delete the mean and std for those variables. Since the distributions of those variables are skewed, the mean and std does not provide much useful info.**

Answer: Following this comment, we now present the median values as the main results in this paragraph. However, because we agree with the Reviewer that the distributions of those variables are skewed, we let the information into brackets about the distribution of these variables, i.e., SD, mean, 1^st^ quartile, and 3^rd^ quartile, for interested readers.

Following this comment, we corrected the manuscript as follows:

*P10-11: “Of the 17,076 hospitalized adult patients with a positive COVID-19 RT-PCR test, 1,908 patients (11.2%) were excluded because of missing data or their young age (i.e. less than 18 years of age). Of the 86 adult patients who received haloperidol at any time during the visit, 47 (54.7%) patients were excluded because they received it more than 48 hours from hospital admission (N=47, 54.7%) or after intubation (N=16, 18.6%). Of the remaining 15,121 inpatients, 39 patients (0.3%) received haloperidol at baseline (i.e., within the first 48 hours of hospital admission) at a median daily dose of 3.0 mg per day (SD=5.2; mean=4.5; first quartile=1.0; third quartile=5.0; range=0.5-20.0 mg) for a median duration of 7.0 days (SD=7.2; mean=8.4; first quartile=2.5; third quartile=12.0; range=1-26). Of these 39 patients, 4 patients (10.2%) had a medication administration by intramuscular injection. Median delay between study baseline and haloperidol initiation was lower than 0 day (SD=1.0; mean=0.6; first quartile=0.0; third quartile=1.0; range=0-2 days) (S1 Figure).*

*First positive COVID-19 RT-PCR tests were obtained after a median delay of 1.2 days (SD=12.7) from study baseline. This delay was significantly but not substantially different between patients receiving or not receiving haloperidol [median in the exposed group=1.0 day (SD=11.2); median in the non-exposed group=1.2 days (SD=12.8); Mood’s median test Chi-square=3.76, p=0.001].*

*Over a median follow-up of 7 days (SD=17.9; mean=13.8; first quartile=1.0; third quartile=22.0; range: 1-98 days), 2,024 patients (13.4%) had a primary end-point event and 10,179 patients (77.6%) were discharged home at the time of study end on May 1st. Patients receiving haloperidol had a median follow-up of 7 days (SD=14.0; mean=10.9; first quartile=4; third quartile=22; range: 1-80 days), while the non-exposed group had a median follow-up of 7 days (SD=17.9; median=13.8; first quartile=1; third quartile=22; range: 1-98 days) [Mood’s median test Chi-square=0.41, p=0.679].”*

**7/ 2. Study Endpoints**

**For the secondary endpoint, for some reason the authors reported the results from the ‘IPW’ instead of the matched results as the primary analysis. I think it should be the results from the match sample.**

Answer: We warmly thank the Reviewer for noticing this typo, which is now corrected.

*P15, L304-307: “Haloperidol use was significantly and negatively associated with the secondary endpoint in the crude, unadjusted analysis (HR, 0.24; 95% CI, 0.13 to 0.44; p<0.001), but this association was not significant in the primary analysis using a univariate Cox regression model in the matched analytic sample (HR, 1.30; 95% CI, 0.74 to 2.28; p=0.355) (Fig 2; Table 2).”*

We warmly thank the Reviewers for their strong help in improving our manuscript.
